# The Evaluation of Telehealth’s Impact on Medicare Annual Wellness Visits and Dementia Diagnosis

**DOI:** 10.1093/geroni/igaf122.378

**Published:** 2025-12-31

**Authors:** Zhang Zhang, Nancy Schoenborn, Katherine Miller, Jennifer Wolff, Daniel Polsky

**Affiliations:** Johns Hopkins Bloomberg School of Public Health, Baltimore, Maryland, United States; Johns Hopkins University School of Medicine, Baltimore, Maryland, United States; Johns Hopkins Bloomberg School of Public Health, Baltimore, Maryland, United States; Johns Hopkins University - School of Public Health, Baltimore, Maryland, United States; Johns Hopkins University, Baltimore, Maryland, United States

## Abstract

**Background:**

Telehealth has emerged as an essential tool in modern healthcare, providing the potential for the early screening and diagnosis of mild cognitive impairment (MCI) and Alzheimer’s Disease and Related Dementias (ADRD). For example, telehealth enhances access to care and streamlines cognitive assessment during Medicare Annual Wellness Visits (AWVs). Our study aims to evaluate the impact of telehealth on AWV uptake using a large population dataset.

**Method:**

We used the data from Traditional Medicare (TM) and Medicare Advantage (MA) plans. We applied a probit regression model to examine the relationship between telehealth adoption and AWV uptake among beneficiaries aged 65 and older continuously enrolled in traditional FFS plans or MA in 2020-2022.

**Results:**

Preliminary analysis using 2020 TM data revealed that 39% of beneficiaries had ever adopted at least one telehealth visit. 7% of the AWVs were delivered via telehealth among those AWV adaptors. There was a significant increase in AWV uptake among telehealth users by 10.2 percentage points to non-telehealth users in the TM plan (p < 0.001). We will extend this analysis to MA beneficiaries, where we anticipate a more pronounced effect. MA likely has greater capabilities to manage health expenditures and provide resources, such as internet access and incentives for preventive care.

**Conclusions:**

Our study underscores the role of telehealth in increasing AWV uptake and facilitating cognitive assessments. Telehealth holds promises for bridging the gaps in cognitive screening and enhancing early detection of MCI and ADRD, thereby supporting timely diagnosis and care planning.

